# Prevalence and Clinical Implications of HPV Infection in Oral Cavity and Oropharynx in HIV+ Men

**DOI:** 10.1155/ijod/7565387

**Published:** 2026-02-03

**Authors:** José Adán Vizcaíno-Reséndiz, Jaime F. Andrade-Villanueva, Luis Felipe Jave-Suárez, Tonatiuh Abimael Baltazar-Díaz, Felipe De Jesús Bustos-Rodríguez, Luis Eduardo Del Moral-Trinidad, Dana Alejandra Figueroa-González, Juan Carlos Vázquez-Limón, Ana Esther Mercado-González, Luz Alicia González-Hernández

**Affiliations:** ^1^ Unidad de VIH, Hospital Civil de Guadalajara “Fray Antonio Alcalde”, InIVIH, Universidad de Guadalajara, Guadalajara, Jalisco, Mexico, udg.mx; ^2^ Centro de Investigación Biomédica de Occidente (CIBO), Instituto Mexicano del Seguro Social (IMSS), Guadalajara, Jalisco, Mexico, imss.gob.mx; ^3^ Departamento de Biología Molecular y Genómica, Instituto de Investigación en Enfermedades Crónico-Degenerativas, Centro Universitario de Ciencias de la Salud, Universidad de Guadalajara, Guadalajara, Jalisco, Mexico, udg.mx; ^4^ Departamento de Anatomía Patológica, Nuevo Hospital Civil de Guadalajara “Juan I. Menchaca”, Guadalajara, Jalisco, Mexico

**Keywords:** α and β HPV infection, HIV+ MSM, oral cavity, oropharynx

## Abstract

**Introduction:**

Human papillomavirus (HPV) is associated with several types of oral lesions (OLs), including oropharyngeal squamous cell carcinoma (SCC), especially in HIV‐positive individuals.

**Objectives:**

To identify the prevalence and clinical implications of the alpha (α) and beta (β) genera of HPV in the oral cavity and oropharynx of HIV‐positive men who have sex with men (MSM).

**Methods:**

A cross‐sectional study from January 2022 to May 2023 was performed. Ninety‐four participants were included; their sexual habits, risk factors, HIV‐1 viral load, and CD4^+^ T‐cell counts were obtained. Exfoliative cytology was performed, and deoxyribonucleic acid (DNA) was extracted from the samples to identify α and β HPV genera through endpoint polymerase chain reaction (PCR). OLs associated with HPV infection were described, and histopathological and immunohistochemical findings for p16 were reported.

**Results:**

A total of 71.3% of the participants were positive for any HPV genus (22.4% α‐HPV and 49.2% β‐HPV). The 6.3% had OLs associated with HPV, principally leukoplakia, and although six of nine samples were p16 positive, none were malignant.

**Conclusion:**

The prevalence of HPV infection in the oral cavity and oropharynx in HIV + MSM was high; however, the associated OLs were infrequent and not malignant. Future studies are necessary to evaluate the clinical relevance of HPV infection and p16‐positive OLs.

## 1. Introduction

Human papillomavirus (HPV) is a non‐enveloped, double‐stranded deoxyribonucleic acid (DNA) virus that infects the basal layer of the epithelium of the skin and mucosa, increasing cell proliferation and viral replication in fully differentiated keratinocytes [1, 2]. HPVs are characterized by their notable tissue specificity and the different pathological conditions they cause. Viral infections usually remain localized and generally remit spontaneously, although they can recur and persist throughout the host’s life [3]. Clinically evident oral cavity lesions may be benign, such as squamous oral papilloma, verrucae vulgaris, condylomata acuminata, and multifocal epithelial hyperplasia [1]. Moreover, some potentially malignant lesions, such as leukoplakia, can present with active HPV infection and progress to squamous cell carcinomas (SCCs) depending on the presence of certain risk factors, high‐risk HPV genotypes, and medical conditions [4].

The L1 gene from HPV encodes the major capsid protein and is well conserved among different HPVs; therefore, its nucleotide sequence can be used for viral classification. A distinct HPV genotype is assigned when the sequence of its L1 gene differs by at least 10% from any other HPV genotype [5]. Based on this, HPVs are grouped into five main phylogenetic genera: alpha (α), beta (β), gamma (γ), mu (μ), and nu (ν), with viruses belonging to different genera having <60% similarity within the L1 gene [3].

It has been reported that HIV‐positive (HIV+) men who have sex with men (MSM) have a prevalence of α‐HPV infection of up to 30%; in addition, the predictors or determinants of oral HPV infection in the context of α‐HPVs have been well characterized [6]. However, clinical features and prevalence in β‐HPV infection have not been well described. β‐HPV genera can be found in the oral mucosa and saliva as asymptomatic infections and have been reported to have a prevalence of 10%–50% [6]. They have also been identified in head and neck carcinomas [7].

Oral cavity and oropharyngeal cancers associated with HPV infection have been classified as a distinct subset compared to HPV‐negative cancers, with relevant differences. HPV‐associated malignancies develop at an early age compared to non‐HPV‐associated cancers, with or without a history of tobacco use or high alcohol consumption. Furthermore, HPV‐positive oral and oropharyngeal cancers have a lower risk of tumor progression and lower mortality rates owing to better responses to chemotherapy/radiotherapy [8, 9].

The gold standard for oral cancer diagnosis is a biopsy. Clinical inspection is a relevant tool for ruling out or identifying malignant and potentially malignant lesions; in this sense, the clinician’s task is to discriminate between reactive lesions or inflammatory changes that can be interpreted as suspicious of malignant neoplasia. Toluidine blue staining is retained in tissues with increased cellular DNA and RNA activity, helping to delimit anatomical sites suspected of malignancy [10]. A positive result is indicated by an intense blue color, whereas a negative result is indicated by a faint blue color or no staining. This in vivo tissue staining technique has a high sensitivity of 92.6%–97.8% and a specificity of 67.9%–100% for identifying malignant or premalignant lesions, making it an effective method as an adjuvant, indicating to the clinician the most appropriate site for biopsy [11, 12].

p16 immunohistochemistry (p16 IHC) is a tool widely used for the identification of high‐risk HPV‐associated lesions. The p16 protein is encoded by the tumor suppressor gene CDKN2A, located on chromosome 9, which acts as an inhibitor of cyclin‐dependent kinases that decelerate the cell cycle by acting at the G1 phase‐S phase checkpoint and inactivating the protein encoded by the retinoblastoma gene (Rb). There is a reciprocal relationship between pRb and p16 expression levels. It has been shown that in the pathogenesis of oral and oropharyngeal cancer, the integration of the viral genetic material into the genome of the host cells and the subsequent expression of the product of the viral protein E7 determine an inactivation of pRb, increasing cellular proliferation [13]. This inactivation corresponds to the overexpression of p16, which can be detected by immunohistochemical techniques with different patterns, revealing the integration of the viral genome into the host cell genome and serving as a surrogate marker of high‐risk lesions associated with HPV infection prone to developing dysplasia and SCC [14–16].

Studies have reported an increased risk of oral cavity and oropharyngeal cancers in people living with human immunodeficiency virus (PLWHIV) compared to HIV‐negative individuals [17]. The development of malignant neoplasms in PLWHIV is more common in the antiretroviral therapy (ART) era because of increased survival and the opportunity to observe the natural history of the HPV oncogenic process over time [18]. Furthermore, Tat and Gp120 HIV proteins increase viral gene expression. They also allow HPV to penetrate the epithelium and infect the *stratum basale* by disrupting cellular tight junctions. Additionally, the HIV Tat protein increases the expression of the HPV E2 protein (which assists in HPV replication), reactivating latent HPV infection, reduces the production of the p53 protein, and increases the expression of HPV proteins E6 and E7, whose oncogenic functions have been well identified [19].

Oropharyngeal carcinoma is the second most common HPV‐related cancer, after cervical cancer [20]. However, nowadays there are no validated screening methods for the timely detection of oral and oropharyngeal cancers, as there are for cervical and anorectal cancer. Thus, it may be a field of opportunity [9]. The development and implementation of validated screening approaches could enable the early identification of precursor lesions and malignancies, facilitate timely interventions, and reduce disease burden, particularly in high‐risk populations such as HIV + MSM. Therefore, this study aimed to identify the prevalence and clinical implications of the α and β genera of HPV in the oral cavity and oropharynx of HIV + MSM, which could help to clarify the importance of oral and oropharyngeal cancer screening, identify premalignant lesions, provide timely treatments, and identify those subjects who require closer monitoring.

## 2. Materials and Methods

### 2.1. Design of the Study and Recruitment of Participants

A cross‐sectional study was conducted from January 2022 to May 2023 at the HIV Unit of Hospital Civil de Guadalajara “Fray Antonio Alcalde,” a reference university hospital in western Mexico. Subjects were invited to voluntarily participate in the study on the same day of their clinic visit at the HIV unit, according to the following inclusion criteria: HIV + MSM ≥18 years old, in control on ART for at least 12 months, CD4^+^ T‐cell count ≥350 cells/μL (which was considered as immunological responders), no history of HPV vaccine, and all participants voluntarily signed the informed consent to participate. The noninclusion criteria were individuals under immunosuppressive or immunomodulator treatment, active bleeding in the oral cavity secondary to local or systemic pathology (blood dyscrasia), or subjects with previous treatments for pre‐existing oral lesions (OLs) in the last 3 months. The exclusion criteria were insufficient, degraded, or contaminated samples or participants’ requests to abandon the study.

Medical and physical examinations were performed, and a questionnaire about the use of illegal drugs, tobacco, or alcohol; exposure to other kinds of drugs; history of sexual activity (beginning of active sexual life, number of partners, and type of sexual relations, whether oral or anal, and frequency of condom use in oral sex); and comorbidities was administered. In addition, the HIV infection stage of the participants was assessed and stratified according to CDC criteria [21].

### 2.2. Histopathological and Immunohistochemical Analyses

All participants underwent dental, oral, and oropharyngeal evaluation to identify the presence or absence of disease and macroscopic lesions in the oropharyngeal and oral cavity. Participants with clinical suspicion of oropharyngeal and OLs associated with HPV infection were screened using BlueDetect toluidine blue staining (following the manufacturer’s instructions). All positive stains and macroscopic lesions were biopsied and sent to the local pathology department for histopathological and immunohistochemical (p16 antibody) analysis.

### 2.3. DNA Extraction From Oral Samples and End‐Point Polymerase Chain Reaction (PCR)

For HPV identification, oral and oropharyngeal samples were obtained through rinses and gargling using Listerine, according to Mendoza and Heath’s mouthwash protocols [22, 23]. Briefly, without 24 h prior tooth brushing and in a fasting state, the participants were requested to gargle for 15 s and rinse for 45 s. Five mL of the sample was recollected in a sterile plastic tube and preserved at −80°C until DNA extraction.

DNA extraction was performed using a protocol for extracting genomic DNA from oral and oropharyngeal samples with the PureLink Genomic DNA mini kit (K182001, DNA extraction kit of Thermo Fisher Scientific, USA). The concentration and purity of the extracted DNA (A260/A280≥1.80) were determined by spectrophotometry using a NanoDrop 2000 (Thermo Fisher Scientific, USA).

To identify the HPV genotypes in each sample, endpoint and nested PCR tests were performed using PGMY and FAP primers, according to the protocol described by Flores‐Miramontes, Forslund, and Gravitt [3, 24, 25]. We used DNA extracted from the CaSki cell line (containing integrated HPV16 DNA) as an HPV‐positive control and FZD2 as a positive human genomic amplification control.

### 2.4. Statistical Analyses

A formal sample size calculation was not performed due to the wide range of prevalence reported in the literature; a sample size of 94 participants was obtained.

Data normality was examined using the D’Agostino and Pearson omnibus test. A descriptive analysis was performed using arithmetic mean/standard deviation (SD) for parametric data and median/interquartile range (IQR) for nonparametric data. The chi‐square test was applied to evaluate associations between clinical, immunologic, virologic, demographic, histopathological, and IHC variables, and PCR results. All statistical tests were two‐tailed; a *p*‐value of ≤ 0.05 was considered statistically significant. The data were analyzed using SPSS version 25.0 and GraphPad Prism version 9.2.0.

## 3. Results

### 3.1. Sociodemographic, Virological, and Immunological Characteristics

A total of 398 subjects were evaluated, of whom 99 met the selection criteria for this study. Five participants were excluded because their rinse samples were degraded. The final study group comprised 94 participants (participant flow diagram).


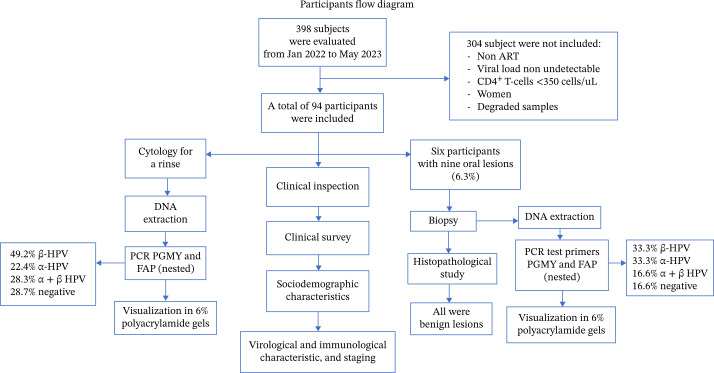


The median age of the participants was 37 years (IQR: 32–46); all were under an ART regimen based on bictegravir/tenofovir alafenamide/emtricitabine, with undetectable HIV‐1 viral load, a median CD4^+^ T lymphocyte count of 588 cells/μL (IQR: 458–759), and median number of years living with HIV of 4 years (IQR: 2–8). Regarding substance consumption habits, we found that 49% of the participants consumed tobacco at the time of the survey, and 63% of the participants consumed alcohol. Illegal drug use was found in 32% of the participants (most frequently, marijuana).

Regarding sexual habits, the beginning of their active sexual life was at a median of 18 years (IQR: 16–20), with a median of 38 sexual partners (IQR: 15–45). About condom use, 84% of participants reported not using condoms during oral sex. The sexual orientation was homosexual in 90%, and just 10% reported being bisexual. Furthermore, the majority (64%) of the participants denied having previous anal or genital lesions associated with HPV (68/94) (Table 1).

**Table 1:** Representation of the sociodemographic, virological, and immunological variables.

		Amplification PCR‐primers FAP/PGMY				
	Total	Positive			Positive for both	Negative
		Total	ß‐HPV	α‐HPV		
*n*	94	67	33	15	19	27

	Median (IQR) or mean (± SD)					

Age (years)	37 (32–46)	37 (31–49)	41 (34–49)	35 (30–56)	34 (27–38)	36 (30–43)
Years living with HIV	4 (2–8)	4 (2–10)	6 (2–14)	3 (2.5–6)	4 (1–11)	5 (3–8)
Beginning of active sexual life	18 (16–20)	17 (16–19)	17 (15–20)	18 (17–20)	17 (15–19)	17 (15–20)
HIV‐1 viral load (cop/mL)	33 ± 8	33 ± 8	31 ± 9	35 ± 7	36 ± 7	32 ± 8
CD4^+^ T lymphocytes (cells/μL)	588 (457–759)	580 (457–759)	544 (376–759)	521 (409–832)	582 (446–987)	566 (81–759)

	Frequency (%)					

Age groups: 20–29 years 30–39 years 40–49 years 50–59 years 60 years and older	13 (14) 40 (43) 25 (27) 11 (12) 5 (6)	5 (5) 21 (22) 12 (13) 7 (7) 3 (3)	3 (3) 10 (11) 13 (14) 6 (6) 1 (1)	1 (1) 9 (10) 2 (2) 2 (2) 2 (2)	6 (6) 9 (10) 2 (2) 1 (1) 2 (2)	4 (4) 11 (12) 8 (8) 2 (2) 0 (0)

Tobacco use	46 (49)	31 (33)	15 (16)	7 (7)	8 (9)	14 (15)
Alcohol consumption	59 (63)	46 (49)	19 (20)	11 (12)	13 (14)	12 (13)
Consumption of drugs	30 (32)	21 (22)	13 (14)	2 (2)	5 (5)	9 (10)
Unprotected oral sex	78 (83)	59 (63)	26 (28)	14 (15)	16 (17)	19 (20)
History of anal or genital lesions due to HPV	34 (36)	24 (25)	13 (14)	3 (3)	8 (9)	10 (11)

Abbreviations: IQR, interquartile range; SD, standard deviation.

### 3.2. Clinical, Histopathological, and Immunohistochemical Characteristics

Two oral pathologists evaluated all participants. Nine oral cavity lesions were observed in six subjects (6.3%, 6/94), comprising leukoplakia (5/9) and papillomatous lesions (4/9). Histopathological analysis of these lesions was compatible with HPV infection in seven of them (77.8%, 7/9). Of note, the mean age of this subgroup was 37 years, with 7.8 years living with HIV and under continuous ARV treatment.

**Figure 1:**
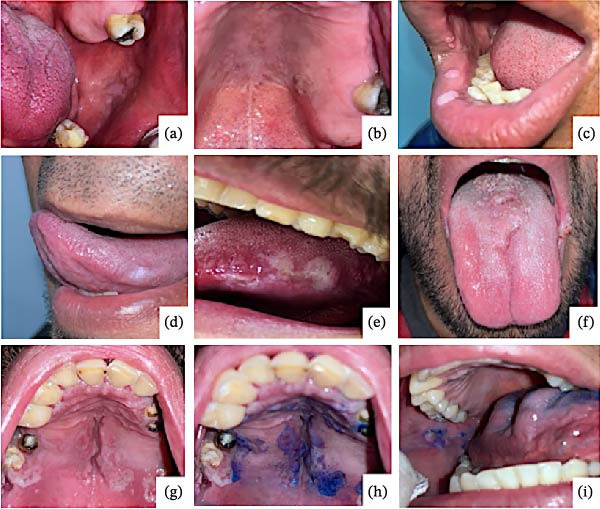
Lesions were identified in the oral cavity of the participants. The lesions observed in the participants were only located in the oral cavity: (a) a whitish lesion with a smooth and raised surface composed of multiple papules on the buccal mucosa, (b) a nodular‐looking lesion with an irregular surface on the hard palate, (c) whitish plaques and papules on the lower and upper labial surface, (d) leukoplakia on the lateral edge of the tongue, (e) leukoplakia with irregular edge and ulcerated surface on the lateral lingual edge, (f) papillomatous surface lesion on the lip commissure and lingual surface, (g) inhomogeneous leukoplakia on the palatal surface, (h) positive toluidine blue staining in the lesion marked with the letter g, and (i) papular lesion with smooth surface and associated white plaque, note that the plaque presented a positivity when stained with toluidine blue. However, the papular zone was observed to be negative.

**Figure 2:**
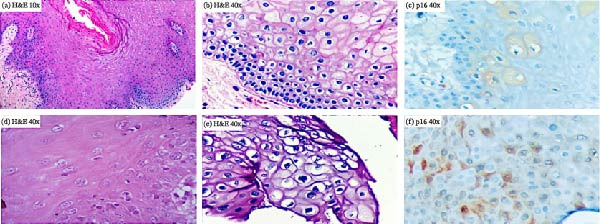
Histopathological findings of lesions identified in the participants. (a), (b), (d), and (e) Images of the histological sections observed in the lesions that were compatible with HPV infection in the subjects, where papillary projections, epithelial acanthosis, koilocytic figures, and nuclear hyperchromatism in suprabasal strata were identified. (c) and (f) Immunohistochemical staining with p16 with intracytoplasmic positivity in keratinocytes of lesions positive for HPV infection. H&E = hematoxylin and eosin staining.

Toluidine blue staining was positive in five of the nine lesions; this characteristic was observed in all plaque lesions (leukoplakias) and not in those with a papillomatous appearance. In all cases, the histopathological studies ruled out malignant or premalignant lesions (Figure 1). In contrast, p16 IHC was positive in six lesions, nonspecific in one, and negative in two. Moreover, most had sporadic and focal patterns (Figure 2). (Supporting information 1 and 2).

### 3.3. Molecular Analysis

Regarding the endpoint PCR, 71.3% (67/94) of the samples were positive for HPV using endpoint and nested PCR with PGMY and FAP primers (which amplify α and ß‐HPV genera, respectively). Of these samples, 22.4% (*n* = 15) corresponded to α‐HPV (PGMY primers), and 49.2% (*n* = 33) were positive for ß‐HPV (FAP primers). Moreover, 19 samples (28.3%) were positive for both primers (indicating α and ß‐HPV co‐infection), and 27 samples (28.7%) were negative for both genera.

With respect to the CDC staging distribution, we found 23 HPV‐positive cases in stage A1, nine in stage A2, two in stage A3, three in stage B1, two in stage B2, and 16 in stage C1. We did not find any significant association between CDC stages and PCR positivity for either PGMY or FAP primers.

In contrast, for the six participants with nine OLs, five were PCR‐positive for any HPV primers, two were positive for the PGMY primer set, and two were positive for FAP primers. Additionally, one case was positive for both primer sets, and one was negative for PGMY and FAP (Table 2). Finally, association tests of clinical, immunological, virological, demographic, histopathological, and IHC data with the presence or absence of HPV were performed; however, we found no statistically significant association (data not shown).

**Table 2:** Relationship of results of the participants with oral lesions, according to histopathological diagnosis, microscopic findings, p16 immunohistochemistry, and molecular results by PCR with both sets of primers.

Case number	Injury by anatomical location	Macroscopic lesion	Reported histopathological findings	Presence of HPV according to microscopy	p16 result	FAP/PGMY positive from oral rinse
P5	Jugal mucosa lingual mucosa	Leukoplakia Leukoplakia	Fibroma Pseudoepitheliomatous hyperplasia	Positive Positive	Positive Positive	FAP positive
P17	Cheek palate	Squamous oral papilloma Squamous oral papilloma	Acanthotic lesion Hyperplastic lesion	Positive Positive	Positive Positive	PGMY positive
P56	Palate tongue	Squamous oral papilloma Squamous oral papilloma	Acute mucositis Acute glossitis	Negative Negative	Negative Negative	Both negative
P73	Lingual mucosa (lateral borders)	Leukoplakia	Acute chronic glossitis	Positive	Nonspecific	Both positive
P75	The lateral edge of the tongue and floor of the mouth	Leukoplakia	Condyloma planum	Positive	Positive	PGMY positive
P97	Labial mucosa	Leukoplakia	Condyloma planum	Positive	Positive	FAP positive

Abbreviations: FAP, primer for amplification of ß‐HPV; P, participant; PGMY, primer for amplification of α‐HPV.

## 4. Discussion

HPV infection has been considered the most common sexually transmitted infection, and its role in the pathogenesis of carcinomas, especially for cervical cancer, has been established [26]. Additionally, it has been recognized as a risk factor for anal, penile, and oropharyngeal cancers [27]; however, there are no guidelines that recommend screening for HPV infection or dysplasia in the oral cavity and oropharynx, as there are for cervical or anal cancers. The purpose of our study was to investigate the prevalence of α‐ and ß‐HPV infections and their clinical implications in the oral cavity and oropharynx in HIV + MSM on ART with HIV control and immunological reconstitution.

According to studies reported by authors such as Cab‐Sánchez, Anaya‐Saavedra, and Méndez‐Martínez [28–30], the frequency of HPV infection in the oral and oropharyngeal cavities in PLWHIV increases to 90%. In this study, we identified a prevalence of HPV infection of 71.3%. The α‐HPV genus was identified in 22.4%, while the β‐HPV genus was found in 49.2%. These findings indicate a higher prevalence of ß‐HPV in our population, which contrasts with the study of Nunes et al. [31], who reported a prevalence of 29.3% of ß‐HPV in the oral cavity. It is worth noting that these differences may be attributed to our methodology, which employed a nested‐endpoint PCR using FAP‐degenerated primers, thereby enhancing both the threshold of detection and the coverage of the β‐HPV genus [3].

Even though β‐HPV could be considered a commensal or pathobiont of oral microbiota, some environmental and local factors may favor processes that facilitate HPV infection, such as chronic inflammation and immunosuppression [31]. In our study, we observed that in ~80% of cases, there was local inflammation related to periodontal diseases at different stages. Additionally, environmental factors such as smoking, heavy alcohol consumption, and illegal drugs were frequently identified among the study group. Furthermore, no vaccine covers the β‐HPV genus, and its impact is unknown, unlike α‐HPV vaccines that prevent cervical cancer in up to 90% of cases [32].

Regarding clinically evident OLs, studies on the Mexican population have reported a prevalence of OLs of 6.9% in subjects over 40 years of age who were under ART [29, 30]. Accordingly, we also found a low prevalence of OLs (6.3%, *n* = 6) in HIV + MSM with a median age of 37 years and on ART based on integrase inhibitors for an average of 7.8 years. Likewise, some ART regimens have been associated with a higher prevalence of OLs secondary to xerostomia, epithelial hyperplasia, and oral ulceration. In this regard, it has been demonstrated that protease inhibitor (PI) regimens, such as lopinavir/ritonavir, can favor HPV infection through damaged cell–cell junctions and enhanced paracellular permeability [18]. In line with this, a higher frequency of OLs in PLWHIV under PIs has been reported (23%), whereas PLWHIV under non‐nucleoside reverse transcriptase inhibitors showed 15%, and participants without ART showed 5% [33]. Notably, this is the first study to evaluate the prevalence of OLs in PLWHIV under a regimen with integrase strand transfer inhibitors, in whom we observed that the prevalence of these lesions was similar to that in subjects without ART.

In our study, the most common OL observed was leukoplakia (56%), followed by papillomatous lesions (44%); these results are in agreement with another Mexican study that reported leukoplakia in 55%, while papillomatous lesions were observed in 12% [29]. This contrasts with Venezuelan research, where leukoplakia (34%) and papillomatous (13%) lesions were less frequent [34]. In one case, the clinical findings suggested the presence of HPV, which was discarded because histopathological, immunohistochemical, and molecular tests were negative for HPV infection. Hence, it is important to analyze the clinically observable lesions with at least a histopathological study that confirms or rules out the diagnosis of a malignant lesion.

Toluidine blue staining was positive in five of nine lesions (56%). Fortunately, none of these were malignant. Interestingly, this technique showed positivity in all plaque lesions, whereas the papillomatous lesions failed the toluidine blue test. Our data reaffirm that, despite the toluidine blue staining in the oral cavity having more than 93% sensitivity, it could show a low specificity (67%) to detect potentially malignant and malignant lesions. Thus, it is important to discriminate between ulcerated areas or zones exposed to chronic trauma, as traumatic lesions can be a source of false positive results [12]. In addition, the histopathological analysis of these lesions was compatible with the signatures of HPV infection (e.g., koilocytic‐like changes) in seven of the nine lesions observed, and the concordance with the endpoint PCR test (HPV positivity) was 100%. However, further studies are required to elucidate the performance of these materials in the oral cavity.

p16 IHC positivity is considered a surrogate marker for the oncogenic process associated with HPV infection, which identifies early premalignant and malignant lesions. Previous reports have documented a strong association between oncogenic HPV infection and overexpression of p16 in oral and oropharyngeal premalignant lesions and cancer. Moreover, the pattern of p16 overexpression could suggest either a malignant or benign lesion [35]. In our study, a limited number of OLs were detected, with p16 IHC positivity observed in 67% (6/9) of cases, with sporadic and focal patterns typically associated with benign lesions. Additionally, clinical, histopathological, and molecular tests agreed with positive results for HPV infection in the OLs, even though p16 IHC was interpreted as nonspecific in one of the lesions; nevertheless, this lesion was positive for both FAP/PGMY, showing abnormalities in histopathological analysis. This is important because different mechanisms through which HPV can promote carcinogenesis, independent of E6 and E7 proteins, have been demonstrated, where p16 IHC results can be negative. Interestingly, this oncogenic mechanism (due to hypermutation) has been described more frequently in the β‐HPV infections than in those associated with α‐HPV [35, 36].

Furthermore, although we obtained positive p16 IHC results, we did not detect dysplastic changes in the lesions that suggested malignancy or SCC. Nevertheless, despite the small number of cases identified in this study, it is essential to provide clinical follow‐up to the people in whom we detected OLs, especially those who were positive for p16, because of the above risk factors, which, combined with the mean age of this subgroup (37 years), make them prone to the possibility of developing malignant lesions [37, 38].

One of the strengths of this study was that all OLs (HPV‐related and not related) were removed by an oral pathologist, which may have prevented the possible progression to malignancy. Second, all participants completed an extended questionnaire about risk factors (“classical,” such as smoking habits alcohol abuse, and others, such as oral sex with multiple partners without protection) at the time of sampling. Third, the participants employed different tests, which allowed us to evaluate the agreement between histopathological, IHC, and molecular techniques.

Nevertheless, our study had several limitations that should be considered. First, a cross‐sectional design cannot determine either persistence or causal role. Second, the study was performed just in subjects with HIV control, with a good immune reconstitution, so 299 subjects had to be excluded (due to not meeting the selection criteria), which could provoke a potential selection bias (e.g., potential selection bias may have occurred due to the inclusion of immunological responders only). Third, the primers used (PGMY and FAP) were designed to amplify conserved regions from α and β HPV genera, respectively; however, these sets of primers can also amplify other HPV genera, albeit with much lower specificity. The knowledge of β genotype data is critical for clinical interpretation (such as high‐risk α genotypes). Thus, next‐generation sequencing is necessary to assess the different HPV genotypes and their diversity in the oropharynx and oral cavity of the same subject. Future studies using next‐generation sequencing will be necessary to clarify the clinical relevance and biological impact of individual HPV genotypes.

## 5. Conclusions

This cross‐sectional study found a high prevalence of HPV DNA detected by PGMY/FAP primers in oral rinses from HIV + MSM on ART (based on integrase inhibitors), with control of HIV and immune reconstitution. Interestingly, the most frequent genus was β‐HPV, which does not have a preventive vaccine.

OLs were identified in a few subjects (principally leukoplakia), but all of them were positive for HPV infection, with histopathological abnormalities, and IHC p16 positive, without malignancy. Future follow‐up studies, especially on those who presented p16 positivity, are necessary to evaluate the final clinical evolution.

## Author Contributions

Conceptualization: José Adán Vizcaíno‐Reséndiz, Luz Alicia González‐Hernández, Jaime F. Andrade‐Villanueva, and Luis Felipe Jave‐Suárez. Data curation: José Adán Vizcaíno‐Reséndiz, Tonatiuh Abimael Baltazar‐Díaz, Luz Alicia González‐Hernández, and Luis Felipe Jave‐Suárez. Formal analysis: Tonatiuh Abimael Baltazar‐Díaz,José Adán Vizcaíno‐Reséndiz, Felipe De Jesús Bustos‐Rodríguez, Juan Carlos Vázquez‐Limón, and Luz Alicia González‐Hernández. Funding acquisition: Jaime F. Andrade‐Villanueva, Luz Alicia González‐Hernández, and Luis Felipe Jave‐Suárez. Investigation: José Adán Vizcaíno‐Reséndiz, Luz Alicia González‐Hernández, Jaime F. Andrade‐Villanueva, Felipe De Jesús Bustos‐Rodríguez, Luis Eduardo Del Moral‐Trinidad, Dana Alejandra Figueroa‐González, and Ana Esther Mercado‐González. Methodology: Tonatiuh Abimael Baltazar‐Díaz, Luis Felipe Jave‐Suárez, Ana Esther Mercado‐González, Felipe De Jesús Bustos‐Rodríguez, Luis Eduardo Del Moral‐Trinidad, Luz Alicia González‐Hernández, and Juan Carlos Vázquez‐Limón. Project administration: Jaime F. Andrade‐Villanueva and Luz Alicia González‐Hernández. Resources: Jaime F. Andrade‐Villanueva, Luz Alicia González‐Hernández, and Luis Felipe Jave‐Suárez. Software: José Adán Vizcaíno‐Reséndiz and Tonatiuh Abimael Baltazar‐Díaz. Supervision: Luz Alicia González‐Hernández and Luis Felipe Jave‐Suárez. Validation: José Adán Vizcaíno‐Reséndiz, Tonatiuh Abimael Baltazar‐Díaz, Luz Alicia González‐Hernández, and Luis Felipe Jave‐Suárez. Visualization: José Adán Vizcaíno‐Reséndiz, Tonatiuh Abimael Baltazar‐Díaz, and Luz Alicia González‐Hernández. Writing – original draft: José Adán Vizcaíno‐Reséndiz, Tonatiuh Abimael Baltazar‐Díaz, and Luz Alicia González‐Hernández. Writing – review and editing: José Adán Vizcaíno‐Reséndiz, Tonatiuh Abimael Baltazar‐Díaz, José Adán Vizcaíno‐Reséndiz, Luis Felipe Jave‐Suárez, Luz Alicia González‐Hernández, Ana Esther Mercado‐González, Felipe De Jesús Bustos‐Rodríguez, Luis Eduardo Del Moral‐Trinidad, Dana Alejandra Figueroa‐González, and Juan Carlos Vázquez‐Limón.

## Funding

This research did not receive any external funding.

## Disclosure

All authors have read and agreed to the published version of the manuscript.

## Ethics Statement

The study followed the Ethical Principles for Medical Research Involving Human Subjects outlined in the Helsinki Declaration of 1975 (as revised in Brazil 2013) and was approved by the Ethics Committee of Hospital Civil de Guadalajara (Office Number HCG/CEI‐1200/22) under protocol code 156/22, dated 18/07/2022.

## Consent

The written informed consent was obtained from each participant before enrollment.

## Conflicts of Interest

The authors declare no conflicts of interest.

## Supporting Information

Additional supporting information can be found online in the Supporting Information section.

## Supporting information


**Supporting Information 1** Database: participants enrolled in the study; where column A shows the order of participants, column B records whether an oral lesion was identified, column C indicates the histopathological diagnosis of the identified lesions, D reports the positivity or negativity of the samples from the previous column to immunohistochemistry for P16, E reports the presence or absence of dysplasia in the tissues analyzed, F reports the positivity to the internal control gene for human DNA in the cells extracted from exfoliative cytology, G indicates results for alpha HPV, H indicates results for beta HPV, I, J, K, L, M, N, O, P, Q, R, S, T, U, V, W, and X indicate the rest of the variables analyzed in the study.

**Supporting Information 2** Supplemental content: information on the position of the primers used, positions of the oligonucleotides, conditions for preparing the PCRs, and an interpretation guide for immunohistochemical staining.

## Data Availability

The data are available from the first author upon request.
