# Supplementary material for: Prevalence and Clinical Implications of HPV Infection in Oral Cavity and Oropharynx in HIV+ Men
Source: Int J Dent. 2026 Feb 3;2026:7565387. doi: 10.1155/ijod/7565387 (PMC12868909; doi:10.1155/ijod/7565387)
Supplement: Supplementary file 2 — Supporting Information 2 Supplemental content: information on the position of the primers used, positions of the oligonucleotides, conditions for preparing the PCRs, and an interpretation guide for immunohistochemical staining. [file IJOD-2026-7565387-s001.docx]

## **Supplemental Content**

## **Primer sequence used**

**Figure 1. View of the positions of primers**


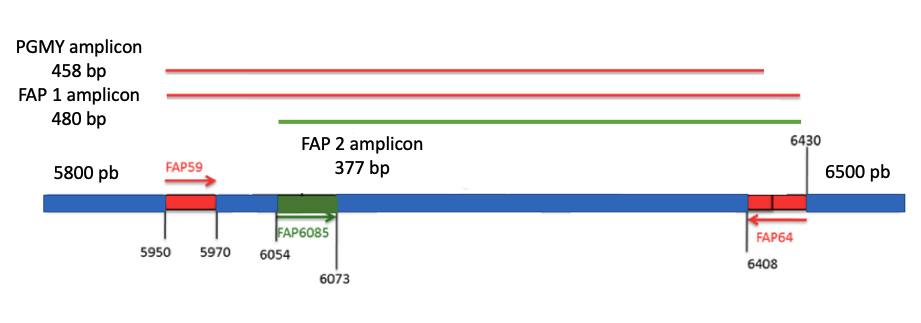


Position of primer pairs in the L1 gene of the open reading frame for HPV amplification. (A) FAP59/64, 458bp products; (B)FAP6085/64, 377 bp product. Modified from Li et al. *(Li et al., 2013).*

## **Table 1. Degenerate nucleotides from FAP primers**

| **Gene** | **Primer design** | **Sequence** | **(bp)** | **Enzyme used** |
| --- | --- | --- | --- | --- |
| **L1** | **FAP59** | **TAA CWG TIG GIC AYC CWT ATT** | **21** | **GoTaq** |
| **L1** | **FAP64** | **CC WAT ATC WVH CAT ITC ICC ATC** | **23** | **GoTaq** |
| **L1** | **FAP85** | **CC WGA TCC HAA TMR RTT TGC** | **20** | **GoTaq** |

**W¯T, C; I¯inosina; Y¯C, T; D¯A, G, T; B¯ G, C, T; H¯A, C, T; V¯A, C, G.**

## **Table 2. Nucleotide sequence for PGMY primers**

| **Gene** | **Primer design** | **Sequence** | **(bp)** | **Enzyme used** |
| --- | --- | --- | --- | --- |
| **L1** | **PGMY11-A** | **GCA CAG GGA CAT AAC AAT GG** | **21** | **GoTaq** |
| **L1** | **PGMY11-B** | **GCG CAG GGC CAC AAT AAT GG** | **21** | **GoTaq** |
| **L1** | **PGMY11-C** | **GCA CAG GGA CAT AAT AAT GG** | **21** | **GoTaq** |
| **L1** | **PGMY11-D** | **GCC CAG GGC CAC AAC AAT GG** | **21** | **GoTaq** |
| **L1** | **PGMY11-E** | **GCT CAG GGT TTA AAC AAT GG** | **21** | **GoTaq** |
| **L1** | **PGMY09-F** | **CGT CCC AAA GGA AAC TGA TC** | **21** | **GoTaq** |
| **L1** | **PGMY09-G** | **CGA CCT AAA GGA AAC TGA TC** | **21** | **GoTaq** |
| **L1** | **PGMY09-H** | **CGT CCA AAA GAA AAC TGA TC** | **21** | **GoTaq** |
| **L1** | **PGMY09-I** | **G CCA AGG GGA AAC TGA TC** | **18** | **GoTaq** |
| **L1** | **PGMY09-J** | **CGT CCC AAA GGA TAC TGA TC** | **18** | **GoTaq** |
| **L1** | **PGMY09-K** | **CGT CCA AAG GGA TAC TGA TC** | **18** | **GoTaq** |
| **L1** | **PGMY09-L** | **CGA CCT AAA GGG AAT TGA TC** | **18** | **GoTaq** |
| **L1** | **PGMY09-M** | **CGA CCT AGT GGA AAT TGA TC** | **18** | **GoTaq** |
| **L1** | **PGMY09-N** | **CGA CCA AGG GGA TAT TGA TC** | **18** | **GoTaq** |
| **L1** | **PGMY09-P** | **G CCC AAC GGA AAC TGA TC** | **18** | **GoTaq** |
| **L1** | **PGMY09-Q** | **CGA CCC AAG GGA AAC TGG TC** | **21** | **GoTaq** |
| **L1** | **PGMY09-R** | **CGT CCT AAA GGA AAC TGG TC** | **21** | **GoTaq** |
| **L1** | **HMB01** | **GCG ACC CAA TGC AAA TTG GT** | **21** | **GoTaq** |

## **Working conditions for carrying out PCRs**

## **Table 3.**

| *PCR reaction for PGMY* | | |
| --- | --- | --- |
| DNA | 1 μl | 100 ng |
| Go Taq master mix | 12.5 μl | 2mM |
| Primer F and R | 1 μl | 5 pmol |
| Nuclease-free water | 10.5 μl |  |

## **Table 4.**

| PCR reaction for FZD2 | | |
| --- | --- | --- |
| DNA | 1 μl | 100 ng |
| Go Taq master mix 2x | 12.5 μl |  |
| Primer pool | 2 μl | 5 pmol |
| Nuclease-free water | 9.5 μl |  |

## **Table 5.**

| *PCR reaction for FAP* | | |
| --- | --- | --- |
| DNA | 1 μl | 100 ng |
| Go Taq master mix 2x | 12.5 μl |  |
| Primer | 2 μl | 5 pmol |
| Nuclease-free water | 9.5 μl |  |

## **Table 6.**

| **Amplification conditions for PGMY** | |
| --- | --- |
| Temperature | Time |
| 95º C | 10 min |
| 95º C  54º C  72º C | 30 seg  30 seg 35 cycles  1 min |
| 72º C | 10 min |

## **Table 7.**

## **Amplification conditions for FZD2**

| Temperatura | Tiempo |
| --- | --- |
| 94º C | 2 min |
| 94º C  56º C  72º C | 30 seg  30 seg 35 cycles  1 min |
| 72º C  4º C | 1 min  1 min |

## **Table 8.**

| **Amplification conditions for FAP** | |
| --- | --- |
| Tempeture | Time |
| 95º C | 10 min |
| 95º C  48º C  72º C | 30 seg  30 seg 35 cycles  1 min |
| 72º C | 10 min |

**Interpretation guide for immunohistochemistry staining**

The CINtec® Histology antibody, anti-p16INK4a (clone E6H4), binds to the human cellular protein p16INK4a (p16). As a cyclin-dependent kinase inhibitor, p16 plays a key role in cell cycle regulation and cellular differentiation. The p16 protein controls the retinoblastoma protein (pRB)-mediated G1-S phase transition and triggers cell cycle arrest in the course of the cellular differentiation process. In normal, terminally differentiated cells, p16 is expressed at low levels typically not detectable by immunohistochemistry (IHC). Research studies have identified strong overexpression of p16 in precancerous and cancerous tissues to be closely linked to the expression of the human papillomavirus (HPV) E7 oncoprotein.

Diffuse staining demonstrates a continuous staining pattern of the basal and parabasal cell layers with or without the staining of the intermediate or intermediate to superficial cell layers as shown below in the images of CIN lesions.

**Figure 2. Positive CINtec Histology status**


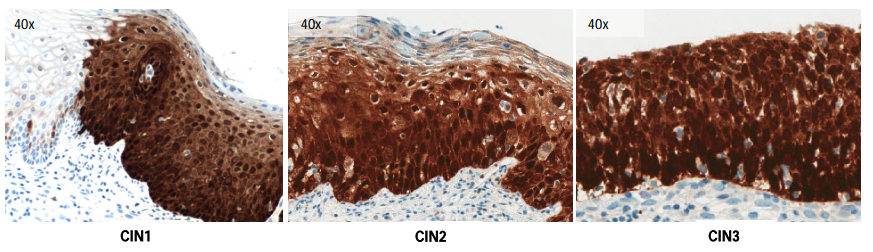


CINtec Histology-stained case examples showing diffuse p16 staining. Final CIN diagnosis for each case is based on the respective H&E-stained slide.

**Figure 3. Negative CINtec Histology status:**


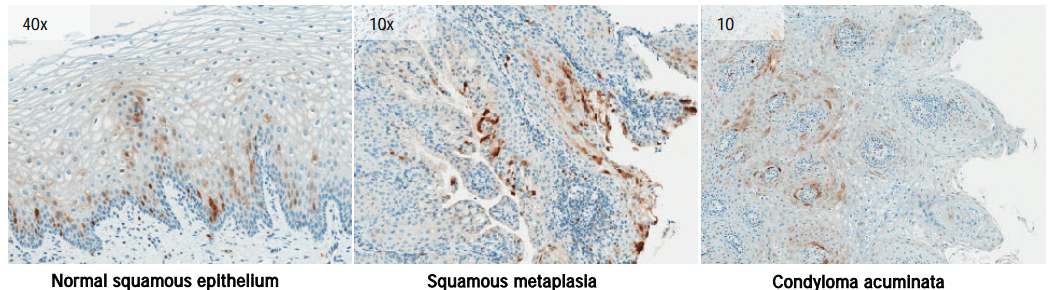


CINtec Histology-stained case examples showing focal p16 staining of single cells and small cell clusters. Final diagnosis for each case is based on the respective H&E-stained slide.

**Figure 4. Negative CINtec Histology status:**


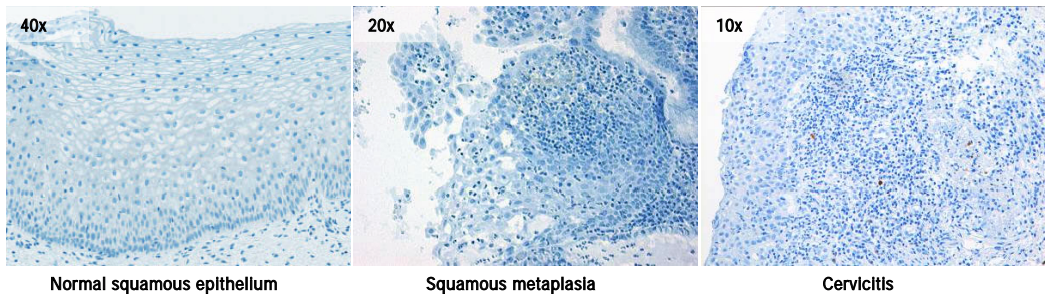


CINtec Histology-stained case examples showing no p16 staining. Final diagnosis is based on the respective H&E-stained slide.

In the following link you will find the page that shows the Classification System for HIV Infections

Link for CDC <https://www.ncbi.nlm.nih.gov/books/NBK219143/>

31. CDC Infección Por VPH Available online: https://www.cdc.gov/hpv/parents/about-hpv-sp.html#:~:text=El VPH%2C o virus del,los 12 años de edad (accessed on 6 September 2024).

32. Greenspan, D.; Canchola, A.J.; MacPhail, L.A.; Cheikh, B.; et al. Effect of Highly Active Antiretroviral Therapy on Frequency of Oral Warts. *Lancet* 2001, *357*, 1411–1412, doi:10.1016/S0140-6736(00)04578-5.

33. María Bravo, I.; Correnti, M.; Escalona, L.; Perrone, M.; Brito, A.; et al. Prevalencia de Lesiones Bucales En Pacientes VIH +, Relación Con Contaje de Células CD4+ y Carga Viral En Una Población Venezolana. *Oral Medicine and Pathology* 2005, *11*, E33–E39.

34. Gabrielli Fregonesi, P.A.; Teresa, D.B.; Duarte, R.A.; et al. P16 INK4A Immunohistochemical Overexpression in Premalignant and Malignant Oral Lesions Infected with Human Papillomavirus. *Journal of Histochemistry & Cytochemistry* 2003, *51*, 1291–1297, doi:10.1177/002215540305101006.

35. Tampa, M.; Mitran, C.I.; Mitran, M.I.; et al. The Role of Beta HPV Types and HPV-Associated Inflammatory Processes in Cutaneous Squamous Cell Carcinoma. *J Immunol Res* 2020, *2020*, 1–10, doi:10.1155/2020/5701639.

36. Sand, L.; Jalouli, J. Viruses and Oral Cancer. Is There a Link? *Microbes Infect* 2014, *16*, 371–378, doi:10.1016/j.micinf.2014.02.009.

37. Kumaraswamy, K.; Vidhya, M. Human Papilloma Virus and Oral Infections: An Update. *J Cancer Res Ther* 2011, *7*, 120, doi:10.4103/0973-1482.82915.

38. Li, J., Pan, Y. Q., Xu, Z.,et al. Improved detection of human papillomavirus harbored in healthy skin with FAP6085/64 primers. *Journal of Virological Methods 2013*, *193*(2), 633–638. <https://doi.org/10.1016/j.jviromet.2013.06.026>

39. Classification System for HIV Infections. Link for CDC <https://www.ncbi.nlm.nih.gov/books/NBK219143/>
